# Exploring Associations between Healthcare Use and Demographics, Pain and Pain Cognitions in People Scheduled for Surgery for Lumbar Radiculopathy: A Cross-Sectional Study

**DOI:** 10.3390/jcm12010388

**Published:** 2023-01-03

**Authors:** Eva Huysmans, Lisa Goudman, Iris Coppieters, Anneleen Malfliet, Wouter Van Bogaert, Jo Nijs, Maarten Moens, Ronald Buyl, Kelly Ickmans, Koen Putman

**Affiliations:** 1 Pain in Motion Research Group (PAIN), Department of Physiotherapy, Human Physiology and Anatomy, Faculty of Physical Education & Physiotherapy, Vrije Universiteit Brussel, Laarbeeklaan 103, 1090 Brussels, Belgium; 2 Department of Physical Medicine and Physiotherapy, Universitair Ziekenhuis Brussel, Laarbeeklaan 101, 1090 Brussels, Belgium; 3 Research Foundation Flanders (FWO), Egmontstraat 5, 1000 Brussel, Belgium; 4 Department of Neurosurgery, Universitair Ziekenhuis Brussel, Laarbeeklaan 101, 1090 Brussels, Belgium; 5 Center for Neurosciences (C4N), Vrije Universiteit Brussel (VUB), Laarbeeklaan 103, 1090 Brussels, Belgium; 6 Stimulus Consortium (Research and Teaching Neuromodulation Uz Brussel), Universitair Ziekenhuis Brussel, Laarbeeklaan 101, 1090 Brussels, Belgium; 7 The Laboratory for Brain-Gut Studies (LaBGAS), Translational Research Center for Gastrointestinal Disorders (TARGID), KU Leuven, Herestraat 49, 3001 Heverlee, Belgium; 8 Interuniversity Centre for Health Economics Research (I-CHER), Department of Public Health (GEWE), Faculty of Medicine and Pharmacy, Vrije Universiteit Brussel, Laarbeeklaan 103, 1090 Brussels, Belgium; 9 Department of Health and Rehabilitation, Unit of Physiotherapy, Institute of Neuroscience and Physiology, Sahlgrenska Academy, University of Gothenburg, 41119 Gothenburg, Sweden; 10 Department of Radiology, Universitair Ziekenhuis Brussel, Laarbeeklaan 101, 1090 Brussels, Belgium; 11 Department of Biostatistics and Medical Informatics, Faculty of Medicine and Pharmacy, Vrije Universiteit Brussel, Laarbeeklaan 103, 1090 Brussels, Belgium

**Keywords:** lumbar radiculopathy, healthcare use, analgesics use, sex, age, socio-economic status, pain intensity, healthcare visits, surgery

## Abstract

This cross-sectional study explored associations between demographics, pain intensity and cognitions on the one hand and healthcare use (HCU) on the other hand in people undergoing surgery for lumbar radiculopathy. HCU during the 2 months preceding surgery was evaluated using a retrospective questionnaire. Demographics included sex, age and level of education and equivalent income. Back and leg pain intensity were evaluated using a visual analogue scale. Pain cognitions were assessed with the Tampa scale of kinesiophobia, the pain catastrophizing scale and the pain vigilance and awareness questionnaire. The sample comprised 120 participants (52% males; 49 years (Quartile (Q)1–Q3: 37.3–57.43)). The number of visits to the general practitioner was associated with sex (incidence rate ratio (IRR) for males = 0.811; *p* = 0.050), pain catastrophizing (IRR = 1.010; *p* = 0.041), pain magnification (IRR = 1.058; *p* = 0.004) and leg pain intensity (IRR = 1.004; *p* = 0.038). The number of neurosurgeon visits was associated with level of education (IRR moderate education = 1.518; *p* = 0.016 (reference: low education)). Receiving zero physiotherapy visits was associated with higher back pain intensity (Beta = 0.018; *p* = 0.028). Highest level of analgesics used was associated with sex (IRR for males = 0.502; *p* = 0.047) and leg pain (IRR = 1.014; *p* = 0.034). Only the association between general practitioner visits and pain magnification remained significant in multivariable analyses (IRR = 1.061; *p* = 0.033). The results suggest a rather indirect relationship between HCU and demographics, pain intensity and cognitions, involving a potential interplay between several patient- and healthcare system-related factors.

## 1. Introduction

Lifetime incidence of low back pain is estimated between 49–70% [1]. It is one of the leading causes of disability [2,3] and responsible for billions of dollars in healthcare expenditure annually [2,3]. A subgroup of people with low back complaints suffers from lumbar radiculopathy (3–5% of the general population [4]), characterized by radiating leg pain [1]. In the latter, surgery is often indicated when symptoms worsen and conservative care fails [4]. Although the majority (±79%) of lumbar decompressive surgeries are anatomically successful [5], 3–36% of the patients experience post-surgical recurrent pain and disability leading to high healthcare use (HCU) [3].

HCU is not only determined by pain [6,7,8] and disability [6,7,8,9], but by different predisposing, enabling and need factors (cf., Andersen’s Behavioral Model of Health Services Use) [10]. As such, pain-related cognitions, which can be categorized as predisposing and/or need factors, may contribute to HCU [11]. A recent systematic review reported evidence for associations between specific pain-related cognitions and HCU outcomes (e.g., between catastrophizing and pain medication use) in people experiencing pain [9]. However, due to inconsistent findings for several pain-related cognitions (e.g., fear-avoidance beliefs and hypervigilance) and HCU measures, future research was recommended to unravel the role of these cognitions in HCU [9]. Moreover, none of the 90 studies in the systematic review considered people with lumbar radiculopathy. If a relationship between pain-related cognitions and HCU is confirmed in people undergoing lumbar decompressive surgery, perioperative interventions specifically targeting maladaptive pain cognitions could be implemented, which could potentially reduce postoperative HCU. This would particularly be relevant as pain-related cognitions are risk factors for an unfavorable outcome following lumbar surgery [12,13,14].

Other enabling and predisposing factors potentially underlying HCU are measures of socioeconomic status (SES) (e.g., equivalent income and level of education), age and sex. Previous research found evidence for a relationship between HCU and SES. However, depending on the healthcare system and HCU measures, either positive or negative associations were reported [15]. Overall, high SES led to a higher probability to consult secondary care, even after adjusting for health need [15,16]. For visits to the general practitioner (GP), on the other hand, low SES was found to be related with more visits [15]. Conflicting results were found for the association between SES and medication use, with some studies reporting higher probabilities to use medication for higher social classes [17,18], and others supporting an inverse relationship [18,19,20]. For age and sex, the literature overall agrees that higher age [19,20] and female sex [17,19,20,21,22] are related to higher levels of healthcare visits and medication use.

The aforementioned associations between patient-related predisposing and need factors and HCU have to the best of our knowledge not been investigated in people with lumbar radiculopathy before. Therefore, the present cross-sectional study aims to explore associations between age, sex, SES, pain intensity and pain cognitions (i.e., kinesiophobia, pain catastrophizing and hypervigilance) and HCU in people with lumbar radiculopathy scheduled for surgery. In line with the evidence in other populations presented above, it is hypothesized that higher age, female sex and higher levels of pain and maladaptive pain cognitions would be associated with higher levels of HCU. In terms of SES, it is expected that lower SES would be associated with more GP visits, but less specialist visits. For the remaining associations investigated in this explorative analysis, no hypotheses could be predetermined based on the existing evidence.

## 2. Materials and Methods

### 2.1. Study Design

This cross-sectional study was reported in accordance with the STROBE (STrengthening the Reporting of OBservational studies in Epidemiology) statement [23]. Participants were initially recruited for a multicenter double-blind randomized controlled trial [24] (registered on ClinicalTrials.gov: NCT02630732). The study was conducted in agreement with the revised Declaration of Helsinki (2013). The protocol was approved by the Ethics Committee of the University Hospital Brussels (B.U.N.143201526926). Baseline data of the randomized controlled trial were used for this cross-sectional analysis.

### 2.2. Participants

Participants were recruited from the University Hospital Brussels (Belgium), AZ Sint Dimpna (Geel, Belgium) and AZ Sint Maarten (Mechelen, Belgium) between March 2016 and April 2019. To be eligible to participate, people had to (1) be scheduled for surgery for (unilateral) lumbar radiculopathy, (2) be able to read and speak Dutch, (3) be between 18 and 65 years of age, and (4) had continued their usual care for 3 weeks before the surgery (i.e., not started any new treatments 3 weeks before the surgery). However, if they already had a habit of using a particular therapy (e.g., medication use or regular therapist visits) from >3 weeks pre-surgery, they could continue to do so. In the participating hospitals no standardized presurgical therapy program was applied at the moment of assessment. People were not eligible if they (1) had symptoms of spinal cord compression, (2) had uncontrolled chronic pain due to a chronic illness other than their low back problems, (3) were diagnosed with rheumatic, neurological or psychiatric disorders or (4) were pregnant or gave birth in the past year. Additionally, participants were asked not to start new treatments during the 3 weeks preceding their scheduled surgery. All participants provided written informed consent before initiating the study.

### 2.3. Outcome Measures

Assessments took place during the week before the surgery. Data were all collected via self-reported (online) questionnaires, except for the participant’s age and sex which were extracted from hospital records. Data collection was executed by an independent researcher not involved in recruitment.

#### 2.3.1. Socio-Economic Status

Two proxies for SES were used: equivalent income and educational level. Educational level was determined by the International Standard Classification of Education (ISCED) and categorized in accordance with the aggregation used by Eurostat [25]. Participants were subdivided into 3 categories: low (ISCED classification 0–2; below or equal to lower secondary level), moderate (ISCED classification 3–4; upper secondary and non-tertiary post-secondary education) or high (ISCED classification 5–8; tertiary education and up) education. Equivalent income was calculated based on the monthly household income and household composition in accordance with the modified Organisation for Economic Co-operation and Development scale [26]. Subsequently, an equivalent income level was assigned to each participant [27]. Low equivalent income was defined as 60% of the median national equivalent income (i.e., at-risk-of-poverty threshold [28]; based on SILC 2018) or lower. Moderate equivalent incomes ranged between 60 and 120% of the median national equivalent income. Equivalent incomes of 120% of the median national equivalent income or higher were categorized as “high”.

#### 2.3.2. Pain Intensity

Pain intensity was assessed using a horizontally oriented visual analogue scale (VAS) ranging from “no pain” (score 0) to “worst imaginable pain” (score 100) [29,30]. Participants were asked to rate their average (last 7 days) low back and leg (symptomatic side) pain separately.

#### 2.3.3. Pain Cognitions

Kinesiophobia was measured with the Tampa scale of kinesiophobia [31] (TSK; Dutch version [32]). This is a self-reported scale for which 17 statements about fear of movement have to be rated on a 4-point Likert scale, resulting in a score range between 17 and 68. The cut-off score for a clinically relevant degree of kinesiophobia is 37/68 [32]. The TSK has good clinimetric properties in people with low back pain [33].

The level of pain catastrophizing was assessed using the self-reported pain catastrophizing scale (PCS; Dutch version) (total score range: 0–52) [34]. The PCS measures 3 related constructs of pain catastrophizing: magnification, rumination and helplessness, for which subscale scores can be calculated [35]. Participants are asked to score 13 pain-related cognitions on a 5-point Likert scale. Scores of 30/52 or higher indicate a clinically relevant degree of pain catastrophizing [36]. The PCS has established clinimetric properties, including good internal consistency, test-retest reliability and proven construct and criterion validity in a variety of populations with pain [35,37,38,39].

The pain vigilance and awareness questionnaire (PVAQ; Dutch version) was used to assess attention to (changes in) pain. This 16-item questionnaire was designed to assess vigilance, awareness, observation and consciousness of pain. All items are rated on a 6-point scale (score range: 0–80). The PVAQ is valid and reliable for use in both populations with pain and pain-free individuals [40,41].

#### 2.3.4. Healthcare Use

Patients were asked to complete a self-reported recall questionnaire concerning their HCU during the preceding 2 months. The questionnaire comprised questions regarding medication use, consultations with healthcare providers and hospitalizations. Recall questionnaires concerning HCU have been found to be feasible and valid for recall periods of up to 6 months [42]. However, to minimize recall bias, participants were instructed to have their agenda with HCU appointments and medication list with them when completing the questionnaire. Additionally, quality of the data was controlled by an independent researcher, and, in case of suspected incompleteness or unclarities, the respective patient was contacted to provide additional input/clarification.

HCU data was subsequently processed into a set of variables of interest for this study. First, the reported medication was subdivided into analgesics and other medication. For each category the number of different drugs used was recorded by counting the number of unique ATC-codes (Anatomical Therapeutic Chemical Classification System) per patient per category. Next, the analgesic level was determined for each type of pain medication according to the World Health Organization’s analgesics ladder [43]. For each participant, the highest level of pain medication taken was determined (no pain medication; level 1 non-opioid analgesics; level 2 weak opioids; level 3 potent opioids) [43]. For visits with healthcare providers, a count variable was created for each healthcare provider category (GP, neurosurgeon, neurologist, other specialized medical doctors, chiropractor, physiotherapist, osteopath, acupuncturist, helplines and other healthcare providers) indicating the total number of visits during the past 2 months with the respective provider.

Information regarding hospitalizations was synthetized by creating a dummy variable for the occurrence of a hospital stay (>1 day) and an additional variable for the total length of stay of all hospitalizations during the past 2 months.

### 2.4. Data Analysis

Statistical analyses were performed using SPSS version 26 (SPSS Inc., Chicago, IL, USA). Descriptive statistics were determined for all demographics and outcome measures. 

The following HCU variables (dependent) were selected for further analysis: (1) highest level of analgesics used (ordinal) and number of visits with the (2) GP, (3) neurosurgeon and (4) physiotherapist (count variables). Visits with other healthcare providers and hospital stays were omitted from further analyses due to their low prevalence in the current sample.

To explore the association between pain intensity and cognitions on the one hand and “highest level of analgesics used” on the other hand, ordinal regression models were executed. For each model, the assumption of proportional odds was checked by interpreting the test of parallel lines. For count variables for visits, first the occurrence of a Poisson distribution was checked. The number of GP and neurosurgeon visits followed a Poisson distribution, but the number of physiotherapy visits did not. The associations between the former 2 and demographics, pain intensity and pain cognitions were therefore analyzed using Poisson regression models. The count variable for physiotherapist visits contained a high number of cases with a count of 0. Therefore, zero-inflated Poisson regression models were used for this dependent variable. All regression models were first executed for each independent factor separately (univariable analyses). Independent variables showing a *p*-value ≤ 0.1 in the univariable analyses of a particular dependent HCU variable were subsequently included in the multivariable enter regression model for that respective HCU variable. Significance level was set at *p* < 0.05.

## 3. Results

### 3.1. Demographics and Descriptives

**Figure 1:**
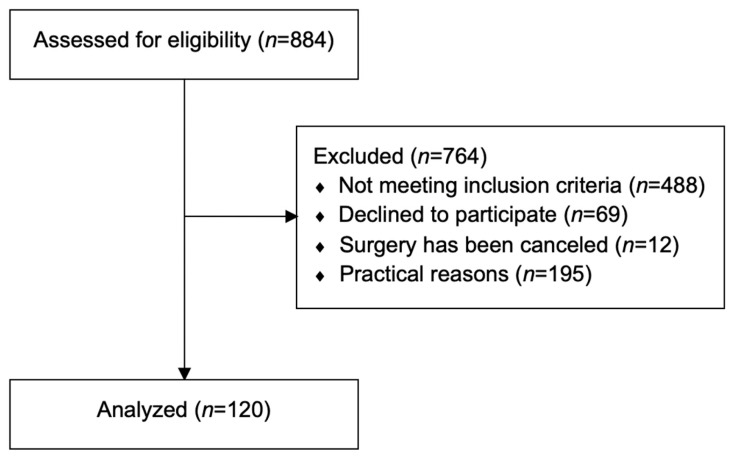
Flowchart.

During recruitment, 884 individuals were screened for eligibility, of which 764 were excluded from study participation (reasons for exclusion: not meeting the predetermined inclusion criteria (*n* = 488), declined to participate (*n* = 69), surgery was canceled (*n* = 12) and other practical reasons (e.g., study protocol not achievable for patient, insufficient time for measurements before surgery, unavailability of assessor; *n* = 195)) (Figure 1). Data of 120 participants (52% males) were available for analyses. Given that none of the continuous data followed a normal distribution, descriptives are presented as medians with corresponding first and third quartiles (Q1–Q3).

Median average low back and leg pain intensity was 45/100 (Q1–Q3: 16.00–66.00) and 55/100 (Q1–Q3: 32.50–77.50), respectively. A median kinesiophobia score of 43/68 (Q1–Q3: 39–47) was reported on the TSK. In terms of pain catastrophizing, the median total score on the PCS was 25/52 (Q1–Q3: 18–32.50). For attention to pain, the median score on the PVAQ was 38/80 (Q1–Q3: 32–48).

Concerning visits with healthcare providers in the 2 months preceding the surgery for lumbar radiculopathy a median number of 3 (Q1–Q3: 2–4) GP, 2 (Q1–Q3: 1–2) neurosurgeon and 0 (Q1–Q3: 0–4) physiotherapist visits were reported. For other healthcare providers both the median and Q1 and Q3 values were below 1. Analgesics were used by 79% of the sample, with 32% using level 1 non-opioid analgesics, 45% using utmost level 2 weak opioid analgesics and 2% using level 3 potent opioids as their highest level of pain medicine. Medication other than analgesics was used by 58% of the sample. Eleven participants (9%) had a hospital stay for various reasons in the 2 months preceding the surgery. None of the participants had more than one hospital stay. Detailed demographics and descriptive statistics are presented in Table 1.

### 3.2. Univariable Regression Analyses

Based on univariable Poisson regression analyses, a significant association with the number of GP visits was found for three independent variables (Table 2): PCS total score (incidence rate ratio (IRR) = 1.010; *p* = 0.041), PCS magnification (IRR = 1.058; *p* = 0.004) and average leg pain intensity (IRR = 1.004; *p* = 0.038). In terms of sex, an IRR for males of 0.811 (*p* = 0.050) was found. The number of visits with the neurosurgeon was significantly associated with level of education (IRR for moderate education = 1.518; *p* = 0.016 (reference category: low education)) (Table 2). Univariable zero-inflation Poisson models for the number of physiotherapy visits found a significant association with average back pain intensity on the zero-inflation model component (B = 0.018; *p* = 0.028) (Table 3). Two independent variables were significantly associated with the highest level of analgesics used based on univariable ordinal regression models (Table 2): sex (IRR for males = 0.502; *p* = 0.047) and average leg pain intensity (IRR = 1.014; *p* = 0.034).

### 3.3. Multivariable Regression Analyses

Only the PCS magnification score (IRR = 1.061; *p* = 0.033) remained significantly associated with the number of GP visits in the multivariable Poisson regression model. None of the independent variables included in the multivariable ordinal regression for the highest level of analgesics used resulted in a significant association. Coefficients for the multivariable models are presented in Table 4. No multivariable analysis was performed for the number of neurosurgeon and physiotherapy visits, because only one independent parameter was found to be related with each of these variables in their respective univariable analyses.

## 4. Discussion

### 4.1. Discussion of the Results

This cross-sectional study explored associations between HCU on the one hand and demographics, pain intensity and pain cognitions on the other hand in people undergoing surgery for lumbar radiculopathy. A negative association was found with the male sex (borderline significance). However, in the multivariable analyses only the association with the PCS magnification subscale remained significant. The number of visits with the neurosurgeon was positively associated with having a moderate level of education compared to low education (univariable analysis). Male sex and leg pain intensity showed, respectively, a negative and positive association with the highest level of analgesics used (univariable analysis). However, both independent variables lost significance in the multivariable model. Lastly, having zero visits with a physiotherapist was found to be positively associated with low back pain intensity (univariable analysis).

Preoperative self-reported levels of kinesiophobia and pain catastrophizing in our sample of people with lumbar radiculopathy are in line with previous research in populations undergoing lumbar surgery [44,45,46,47]. Compared to non-surgical samples with chronic low back pain, the values for catastrophizing and kinesiophobia appear to be somewhat higher in the present preoperative sample [48,49]. PVAQ scores were similar to those of people with chronic spinal pain [48]. These figures suggest that people scheduled for lumbar surgery can potentially benefit from cognitive-behavioral interventions that are effectively targeting maladaptive cognitions in people with chronic (spinal) pain [44,50]. Moreover, maladaptive cognitive and emotional factors are found to be related to acute postoperative pain intensity [51,52,53] and the development of persistent postoperative pain [12,13,14,53]. This indicates the importance of assessing the presence of maladaptive pain-related cognitions and considering perioperative interventions specifically targeting the latter [54].

Univariable analyses supported an association between male sex and a lower number of GP visits and lower likelihood of using a higher level of analgesics. This is in line with previous research on sex-differences in HCU, confirming the assumption that the female sex is positively related with HCU [17,19,20,21,22]. Several hypotheses may explain this consistent finding. Women may show a poorer perceived health status and have less social stigma to admit they are having pain/symptoms [21]. Additionally, women may be at higher risk for experiencing (higher levels of) pain due to higher sensitivity levels related to the female sex [55]. Although it should be mentioned that these associations became non-significant in the multivariable analyses, suggesting an indirect relationship between sex and HCU. Previously reported associations between measures of SES and HCU [15,17,18,19,20,22] could only be confirmed for level of education and number of visits to the neurosurgeon, with individuals with a moderate level of education reporting more visits to the neurosurgeon compared to those with low education. Potentially, higher educated people show better health literacy and knowledge of the healthcare system, which facilitates finding their way to the appropriate specialized medical doctor [15]. In addition, higher education is often related to more prestige and an extensive social network [15], which could again facilitate access to care [15] and contribute to the determination of the patient’s care trajectory, including the decision to have surgery or to get a second opinion.

According to the univariable analyses, leg pain intensity, rather than low back pain intensity, was associated with more GP visits and using a higher level of pain medication. This could be explained by the fact that in patients with lumbar radiculopathy, leg pain is often perceived as worse and more discomforting as compared to back pain [1], which is also reflected by the results on the VAS for both locations. Nevertheless, these associations lost significance in the multivariable analyses. Higher low back pain intensity, on the other hand, appeared to be associated with not having physiotherapy visits, however, with a very low effect size (zero-inflation Poisson B = 0.018). This might not be surprising, as those who did not consult a physiotherapist to alleviate their pain symptoms during the 2 months preceding surgery may indeed show higher pain intensity levels in the week before surgery.

In terms of pain cognitions, pain catastrophizing (particularly the magnification construct) was significantly related to the number of GP visits. Moreover, this was the single independent factor that remained significant in the multivariable analysis. A recent systematic review found inconclusive evidence for a potential association between catastrophizing and number of healthcare visits in people experiencing pain [9]. However, in the few studies investigating the relationship between catastrophizing and the probability to have a consultation, significant multivariable associations were reported [6,9,56]. People who catastrophize more may also perceive their symptoms as more threatening, leading to a higher propensity to consult a healthcare provider [9]. The studies included in the systematic review investigated the relationship between catastrophizing and the number of visits with healthcare providers in general and not GP visits in particular [9]. In the present analyses, pain catastrophizing was only associated with the number of GP visits and not with the number of neurosurgeon or physiotherapy visits. Although the Belgian healthcare system does not apply a formal gate keeper role for the GP [57], many people primarily consult their GP, who could then refer them to other care providers if needed [58]. In that sense, the relationship between pain catastrophizing and GP visits might indeed be more direct compared to the one with consultations with other healthcare providers. Based on these findings combined with the already existing evidence, it could carefully be speculated that interventions targeted at maladaptive pain cognitions (e.g., pain catastrophizing) may be able to reduce excessive HCU and related costs. However, based on this exploratory analysis, no conclusions can be made on causality.

In contrast with previous literature on people experiencing pain [9], we could not confirm a relationship between analgesic use and pain catastrophizing in people with lumbar radiculopathy. Furthermore, for kinesiophobia and hypervigilance, no association with level of pain medication used was found. A possible explanation might be the fact that opioids (level 2 and 3) are often only prescribed to bridge the period between the last presurgical consultation and the surgery. In this case, the advice of the surgeon to take the prescribed analgesics might play a more important role than the patient’s pain cognitions. However, presence of maladaptive pain cognitions is also a positive predictor for opioid prescription [59]. Additionally, previous studies confirming a relationship between pain cognitions and analgesics use all included people with chronic pain [9], who may display different analgesic use patterns than the presurgical population investigated here.

Overall, the inconsistencies between the findings in people with chronic (non-specific) pain and the present sample regarding a potential association between HCU and pain cognitions may also be explained by the fact that the present sample received a clear diagnosis for their symptoms and were scheduled for surgery intended to relieve the latter. People with chronic non-specific pain often do not get a clear explanation for their symptoms, wherefore they might show medical shopping behavior to get an answer to their questions and solution for their symptoms [60], which may also drive them into medication misuse [61].

### 4.2. Strengths and Limitations

To our knowledge this is the first study exploring the association between HCU and demographics, pain intensity and cognitions in people undergoing surgery for lumbar radiculopathy. The use of well-established outcome measures and appropriate analyses for the distributions and characteristics of the data contributes to the validity of the results. Other study strengths include compliance with the STROBE statement and a priori study registration. In addition, some limitations must be considered. The present study comprised an analysis of baseline data of a large multicenter randomized controlled trial (*n* = 120), implying that the study may not have been sufficiently powered for the current research questions, as the original sample size calculation was not based on the present analyses. However, the objective of this study has a clear explorative character, aiming to deliver the first indication of possible associations between the reported outcome measures and to provide a basis for further research. Next, to not overly decrease the power of the study, after thorough consideration, a number of variables were selected to be included in the regression analyses. Other choices in this selection (e.g., duration of symptoms, pain phenotype, or pain acceptance) might have resulted in different results. Furthermore, given that this multicenter study was performed solely in Belgium, the direct application potential of the results to different healthcare systems may be limited. Still, it provides a preliminary basis for future research in this area and for the potential development of innovative therapy approaches targeting factors related to HCU or management policies to be investigated in future studies. Last, based on the present explorative cross-sectional study, no conclusions can be made about causal interactions between the outcomes.

## 5. Conclusions

Significant associations were found between HCU outcomes on the one hand and leg and back pain intensity, pain catastrophizing (magnification), sex and level of education on the other hand in people scheduled for surgery for lumbar radiculopathy. Although based on this explorative analysis no conclusions can be made on causality, the results suggest an indirect association between HCU and demographics, pain intensity and cognitions, involving a potential interplay between several patient and healthcare system-related factors.

## Figures and Tables

**Table 1:** Descriptives of demographics, pain intensity, pain cognitions and healthcare use during the past 2 months in people undergoing surgery for lumbar radiculopathy (*n* = 120).

Topic	Variables ^1^	Descriptives ^2^
Demographics	Age (years)	49.16 (Q1–Q3: 37.3–57.43)
	Sex	62 (52%) males 58 (48%) females
	Equivalent income (*n* = 113)	
	Low equivalent income	34 (30%)
	Moderate equivalent income	57 (50%)
	High equivalent income	22 (20%)
	Level of education	
	Low level of education	35 (29%)
	Moderate level of education	48 (40%)
	High level of education	37 (31%)
Pain	Average VAS back (/100)	45.00 (Q1–Q3: 16.00–66.00)
	Average VAS leg (/100)	55.00 (Q1–Q3: 32.50–77.50)
Pain cognitions	TSK (/68)	43.00 (Q1–Q3: 39.00–47.00)
	TSK ≥ 37/68	106 (88%)
	PCS (/52)	25.00 (Q1–Q3: 18.00–32.50)
	PCS ≥ 30/52	40 (33%)
	Rumination subscale (/16)	10.00 (Q1–Q3: 7.00–12.00)
	Magnification subscale (/12)	4.00 (Q1–Q3: 3.00–6.00)
	Helplessness subscale (/24)	11.00 (Q1–Q3: 8.00–16.00)
	PVAQ (/80)	38.00 (Q1–Q3: 32.00–48.00)
HCU	GP visits	3 (Q1–Q3: 2–4)
	Neurosurgeon visits	2 (Q1–Q3: 1–2)
	PT visits	0 (Q1–Q3: 0–4)
	Analgesics use ^3^	95 (79%)
	Level 1 analgesics (*n* = 95)	73 (77%)
	Level 2 analgesics (*n* = 95)	54 (57%)
	Level 3 analgesics (*n* = 95)	2 (2%)
	Opioid use	56 (47%)
	Highest level of analgesics	None: 25 (21%) Level 1: 39 (32%) Level 2: 54 (45%)Level 3: 2 (2%)
	Other medication use	Yes: 69 (58%) No: 51 (42%)
	Hospital stay ^4^	11 (9%)
	Length of stay	4 (Q1–Q3: 3–10)

^1^ If data were not available for the complete sample (*n* = 120), then the number of cases for which the data was available is mentioned between brackets (*n* =). ^2^ Given that none of the continuous data followed a normal distribution, descriptives were presented as medians with corresponding first and third quartiles (Q1–Q3). Categorical data were presented as counts with corresponding percentages. ^3^ Analgesics were categorized according to the World Health Organization’s Analgesics Ladder. Level 1: Non-opioid analgesics; level 2: weak opioids; level 3: potent opioids. ^4^ Hospital stay is presented as a dummy variable (yes/no). None of the participants had more than one hospital stay in the past 2 months. Abbreviations: n: number of cases; Q: quantile; VAS: visual analogue scale; TSK: Tampa Scale for Kinesiophobia; PCS: Pain Catastrophizing Scale; PVAQ: Pain Vigilance and Awareness Questionnaire; HCU: healthcare use; GP: general practitioner; PT: physiotherapist.

**Table 2:** Univariable analyses for associations between HCU (number of GP and neurosurgeon visits and highest level of analgesics used) and demographics, pain intensity and cognitions.

	Poisson Regression for GP Visits				Poisson Regression for Neurosurgeon Visits				Ordinal Regression for Highest Level of Analgesics Used			
Independent Variables	Exp (B) IRR	95% CI	SE	*p*	Exp (B) IRR	95% CI	SE	*p*	Exp (B) IRR	95% CI	SE	*p*
Sex												
Male	0.811	0.658–1.000	0.107	0.050	1.079	0.823–1.414	0.138	0.583	0.502	0.254–0.991	0.348	**0.047**
Female ^1^												
Age	0.995	0.986–1.004	0.005	0.276	0.998	0.987–1.010	0.006	0.756	0.982	0.953–1.011	0.015	0.224
Equivalent income												
High income	0.933	0.679–1.283	0.162	0.671	0.748	0.484–1.156	0.222	0.191	1.148	0.427–3.087	0.510	0.787
Moderate income	0.998	0.780–1.277	0.126	0.988	1.049	0.768–1.432	0.159	0.765	1.232	0.547–2.773	0.404	0.606
Low income ^1^												
Level of education												
High level	0.775	0.591–1.016	0.138	0.065	1.158	0.794–1.689	0.193	0.445	1.382	0.576–3.317	0.439	0.460
Moderate level	0.880	0.688–1.126	0.126	0.309	1.518	1.080–2.134	0.174	**0.016**	1.778	0.786–4.021	0.418	0.168
Low level ^1^												
TSK	1.013	0.996–1.031	0.009	0.130	1.002	0.980–1.025	0.011	0.852	1.036	0.978–1.097	0.029	0.220
PCS total score	1.010	1.000–1.020	0.005	**0.041**	1.006	0.993–1.019	0.007	0.364	1.018	0.984–1.052	0.016	0.287
PCS magnification	1.058	1.018–1.101	0.020	**0.004**	1.008	0.957–1.061	0.026	0.767	1.046	0.916–1.195	0.066	0.497
PCS rumination	1.016	0.987–1.045	0.014	0.284	1.024	0.987–1.062	0.019	0.212	1.076	0.982–1.178	0.046	0.115
PCS helplessness	1.018	0.999–1.037	0.010	0.068	1.009	0.985–1.034	0.012	0.455	1.019	0.956–1.085	0.031	0.554
PVAQ	1.004	0.995–1.013	0.005	0.362	1.001	0.990–1.013	0.006	0.827	1.004	0.975–1.035	0.015	0.767
Average VAS back	1.003	0.999–1.007	0.002	0.114	1.001	0.996–1.006	0.003	0.751	1.006	0.993–1.019	0.006	0.361
Average VAS leg	1.004	1.000–1.008	0.002	**0.038**	1.000	0.995–1.005	0.003	0.960	1.014	1.001–1.027	0.006	**0.034**

^1^ Reference category; Abbreviations: HCU: healthcare use; Exp (B): exponentiated regression coefficient = IRR: incidence rate ratio; CI: confidence interval; SE: standard error; *p*: *p*-value; TSK: Tampa Scale for Kinesiophobia; PCS: Pain Catastrophizing Scale; PVAQ: Pain Vigilance and Awareness Questionnaire; VAS: Visual Analogue Scale. Significant *p*-values (*p* < 0.05) are highlighted in bold.

**Table 3:** Univariable Zero Inflation Models for the association between the number of physiotherapy visits and demographics, pain intensity and cognitions.

	Count Model Coefficients			Zero-Inflation Model Coefficients		
Independent Variables	Estimate	SE	*p*	Estimate	SE	*p*
Sex						
Male Female ^1^	0.015	0.201	0.940	−0.425	0.419	0.310
Age	0.005	0.008	0.518	0.006	0.018	0.735
Equivalent income						
High income	0.167	0.246	0.496	−0.414	0.611	0.498
Moderate income	0.173	0.209	0.408	−0.147	0.504	0.771
Low income ^1^						
Level of education						
High level	−0.330	0.248	0.183	−0.783	0.546	0.152
Moderate level	0.111	0.243	0.646	−0.287	0.539	0.595
Low level ^1^						
TSK	−0.014	0.019	0.436	−0.019	0.034	0.571
PCS total score	0.000	0.012	0.995	−0.001	0.020	0.973
PCS magnification	0.012	0.043	0.782	0.038	0.080	0.640
PCS rumination	0.002	0.029	0.934	−0.055	0.057	0.330
PCS helplessness	−0.005	0.022	0.831	0.014	0.038	0.708
PVAQ	0.005	0.009	0.555	−0.017	0.018	0.336
Average VAS back	0.001	0.004	0.833	0.018	0.008	**0.028**
Average VAS leg	0.000	0.004	0.936	−0.002	0.008	0.781

^1^ Reference category. Abbreviations: SE: standard error; *p*: *p*-value; TSK: Tampa Scale for Kinesiophobia; PCS: Pain Catastrophizing Scale; PVAQ: Pain Vigilance and Awareness Questionnaire; VAS: Visual Analogue Scale. Significant *p*-values (*p* < 0.05) are highlighted in bold.

**Table 4:** Multivariable analyses for the association between HCU (number of GP visits and highest level of analgesics used) and demographics, pain intensity and cognitions.

Independent Variables	Exp (B) IRR	95% CI	SE	*p*
Poisson regression for number of GP visits				
Sex				
Male	0.846	0.669–1.089	0.119	0.161
Female ^1^				
Level of education				
High education	0.847	0.635–1.131	0.147	0.262
Moderate education	0.922	0.717–1.186	0.128	0.527
Low education ^1^				
PCS				
PCS magnification	1.061	1.005–1.120	0.028	**0.033**
PCS helplessness	0.986	0.960–1.014	0.014	0.323
Average VAS leg	1.002	0.998–1.007	0.002	0.287
Ordinal regression for highest level of analgesics used				
Sex				
Male	0.604	0.293–1.247	0.370	0.173
Female ^1^				
Average VAS leg	1.010	0.997–1.024	0.007	0.140

^1^ Reference category. Abbreviations: HCU: healthcare use; CI: confidence interval; SE: standard error; Exp (B): exponentiated regression coefficient = IRR: incidence rate ratio; *p*: *p*-value; PCS: Pain Catastrophizing Scale; VAS: Visual Analogue Scale. Significant *p*-values (*p* < 0.05) are highlighted in bold.

## Data Availability

Any requests (including research plan) to obtain the de-identified data of this study can be send to the corresponding author and will be evaluated within the research consortium. In case the request is deemed appropriate, the data can be shared for further research purposes.
